# Integrated crop management practices for maximizing grain yield of double-season rice crop

**DOI:** 10.1038/srep38982

**Published:** 2017-01-12

**Authors:** Depeng Wang, Jianliang Huang, Lixiao Nie, Fei Wang, Xiaoxia Ling, Kehui Cui, Yong Li, Shaobing Peng

**Affiliations:** 1 National Key Laboratory of Crop Genetic Improvement, MOA Key Laboratory of Crop Ecophysiology and Farming System in the Middle Reaches of the Yangtze River, College of Plant Science and Technology, Huazhong Agricultural University, Wuhan, Hubei 430070, China

## Abstract

Information on maximum grain yield and its attributes are limited for double-season rice crop grown under the subtropical environment. This study was conducted to examine key characteristics associated with high yielding double-season rice crop through a comparison between an integrated crop management (ICM) and farmers’ practice (FP). Field experiments were conducted in the early and late seasons in the subtropical environment of Wuxue County, Hubei Province, China in 2013 and 2014. On average, grain yield in ICM was 13.5% higher than that in FP. A maximum grain yield of 9.40 and 10.53 t ha^−1^ was achieved under ICM in the early- and late-season rice, respectively. Yield improvement of double-season rice with ICM was achieved with the combined effects of increased plant density and optimized nutrient management. Yield gain of ICM resulted from a combination of increases in sink size due to more panicle number per unit area and biomass production, further supported by the increased leaf area index, leaf area duration, radiation use efficiency, crop growth rate, and total nitrogen uptake compared with FP. Further enhancement in the yield potential of double-season rice should focus on increasing crop growth rate and biomass production through improved and integrated crop management practices.

Rice is the staple food for more than half of the world’s population and for more than 65% of the China’s population^1^,^2^. Increasing world rice production in a sustainable manner is vital for ensuring global food security^3^. Global crop production can be increased by expanding the area of croplands, increasing crop yield, and increasing multiple cropping index^4^. Cropland expansion is not feasible because of urbanization and environmental concerns such as biodiversity loss and greenhouse gas emission^4^. It is essential to maintain the increase of rice yield at an annual rate of 1.5%^5^ and at the same time to increase the harvest frequency of existing croplands^4^ in order to keep pace with the food demand of the growing human population.

Grain yield can be increased by breeding new rice varieties with greater yield potential and by improving crop and resource management to enhance actual farm yields^6^,^7^. Optimum crop management especially nutrient management has proven to be highly effective in improving rice grain yield^7^,^8^. Other management practices such as planting methods and plant density, quality of seeds and seedlings, and irrigation regime can also affect grain yield to some extend^9^,^10^,^11^. Qin *et al*. argued that testing single component of management practices independently may not capture the impact a holistic package would have on enhancing rice grain yield^12^. Ladha *et al*. stated that closing the yield gap is becoming increasingly difficult to achieve by using a component technology in isolation^13^. A more integrated approach involving nutrients, water, and other agronomic management factors will allow the maximization of rice grain yield. Furthermore, simultaneously applying a number of the best compatible individual technologies could maximize overall benefits to farmers. Depending on the need and profitability of new technologies, farmers generally integrate new technologies with existing farmers’ practice (FP), which has been referred to as integrated crop management (ICM) or best management practices^13^. Several recent studies have reported greater yield improvement with ICM compared with individual crop production factor^10^,^12^,^14^.

In the subtropical climates, rice can be grown up to two times per year on the same field. In the subtropical environment of Hubei province in China, for example, double-season rice cropping is usually practiced with an early-season crop from April to July and a late-season crop from July to October^15^. The wide adoption of double-season rice systems in both China and elsewhere in Asia increases multiple cropping index and thus contributes substantially to global rice supply^4^. However, the area of double cropping rice has decreased substantially in the last decade in China due to the dramatic increase in labor cost and low grain yield^15^,^16^.

Grain yield of single-season rice crop is higher than that of double-season rice crop^17^. Within the double-season rice cropping system, the early-season rice has lower grain yield than the late-season rice^12^,^15^. The relatively lower yield under early season mainly resulted from slower crop growth during the vegetative phase, which was caused by lower temperature. Reduction in grain filling period due to higher temperature was also responsible for lower grain yield in the early-season rice^12^. Wu *et al*. demonstrated that grain yield of double-season rice can be increased with improved nitrogen (N) management and proper plant density, especially for the early-season rice^15^. It is necessary to determine if ICM can further increase grain yield of double-season rice crop.

Grain yield, radiation use efficiency (RUE), and N use efficiency (NUE) under various crop management practices have been intensively studied for single-season rice crop in China^18^,^19^,^20^. However, relatively little is known about yield performance, yield attributes, and resource use efficiency of double-season rice crop under ICM. Objectives of this study were to (i) compare grain yield and RUE between ICM and FP, (ii) determine maximum grain yield of double-season rice crop in central China, and (iii) identify the traits for improving yield potential of double-season rice.

## Results

### Climatic condition

There was relatively small difference in seasonal average daily minimum and maximum temperatures between the early- and late-season rice (Table 1). However, temperature displayed an increasing trend in the early season, but a decreasing trend in the late season from transplanting to maturity. There was also small difference in seasonal average daily minimum and maximum temperatures between 2013 and 2014. However, higher average temperature was observed in 2013 than in 2014 in the early-season rice from flowering to maturity and in the late-season rice from transplanting to panicle initiation. The opposite was true in the late-season rice from flowering to maturity. Average temperature from panicle initiation to flowering was relatively stable across the two seasons and the two years. There was no clear difference in average daily solar radiation between the early and late seasons. Growing period from flowering to maturity generally had lower average daily solar radiation than other growing periods. Seasonal average daily solar radiation in 2013 was higher than that in 2014 (Table 1).

### Crop growth and development

Relatively small difference in duration from transplanting to flowering was observed across seasons and years (Table 2). The early-season rice had 7 to 9 d longer duration in the seedbed than the late-season rice, whereas the late-season rice had 14–21 d longer duration in the ripening phase (from flowering to maturity) than the early-season rice. Overall, the growth duration of the late season was longer than that of the early season, especially in the main field from transplanting to maturity. Longer growth duration of the late season compared with the early season was mainly due to the difference in the duration of the ripening phase (Table 2).

### Grain yield and its attributes

**Figure 1:**
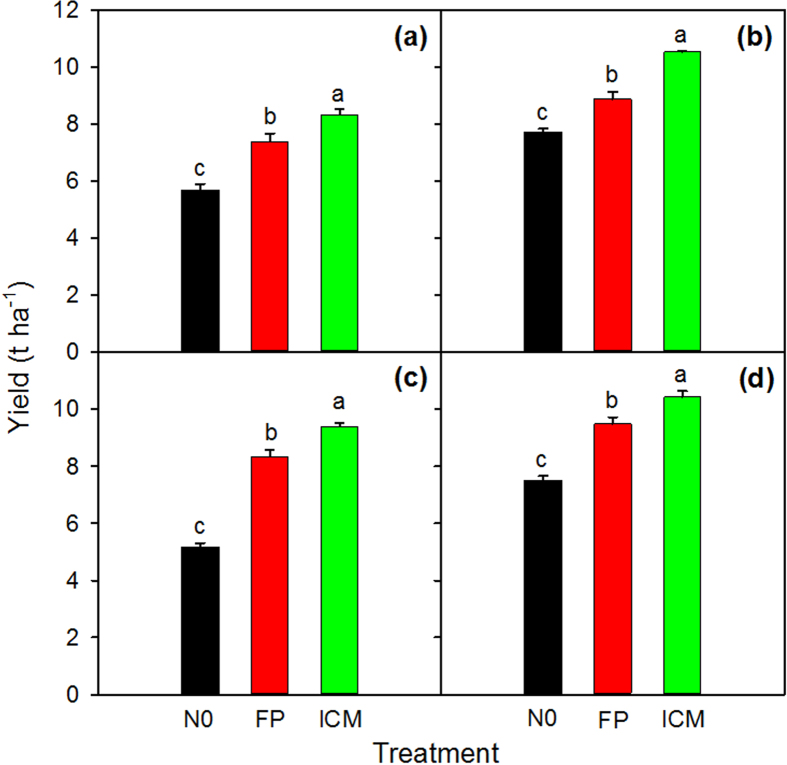
Grain yield in early (**a**) and late (**b**) seasons in 2013, and in early (**c**) and late (**d**) seasons in 2014. Different lowercase letters denote statistical differences between treatments of each season according to LSD test (0.05). Error bars represent ± 1 s.e. (n = 4, standard error of four replications).

Crop management treatments had a significant effect on grain yield in both seasons in the two years (Fig. 1). On average, grain yield in ICM was 12.8% and 14.1% higher than that in FP in the early and late seasons, respectively. Grain yields of ICM and FP were 1.60–3.45 t ha^−1^ higher than that of zero-N control (N0). The late-season rice produced 40.3% higher grain yield than the early-season rice in N0, but only 16.9–18.3% higher grain yield than the early-season rice in FP and ICM (Fig. 1). There was a small and inconsistent difference in grain yield between 2013 and 2014.

Higher grain yield of ICM over FP was mainly attributed to higher spikelets per m^2^ (i.e. sink size), which was caused by the difference in panicles per m^2^ between the two treatments (Table 3). Sink size of ICM was 10.5–18.7% and 18.5–19.9% higher than that of FP in early and late seasons, respectively. At the same time, ICM had 16.3–61.7% and 36.7–54.2% more panicles per m^2^ than FP in early and late seasons, respectively (Table 3). Higher grain yield of late over early season was also due to higher sink size. The difference between the two seasons in sink size was attributed to the difference in panicle size (i.e. spikelets per panicle) instead of panicle number. The sink size of the late-season rice was 19.0% higher than that of the early-season rice, and the panicle size of the late-season rice was 35.6–46.7% higher than that of the early-season rice (Table 3). Grain filling percentage and 1000-grain weight were not responsible for the yield differences between ICM and FP or between the two seasons (Table 3). Average across seasons and years, daily grain yield of ICM and FP was 105.6 and 93.1 kg ha^−1^ d^−1^, respectively (Table 4). There was no consistent difference in daily grain yield between the early and late seasons. Daily grain yield was higher in 2014 than in 2013, except for N0 in the early season (Table 4).

**Figure 2:**
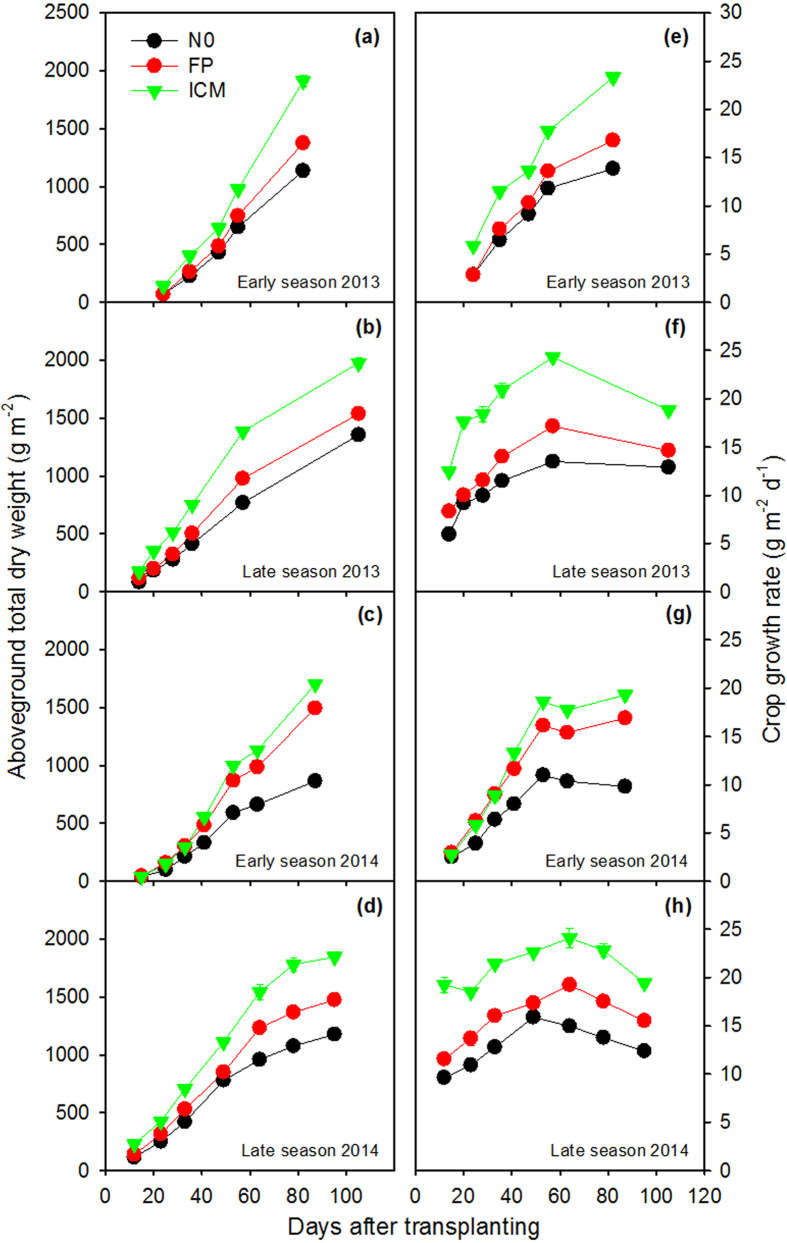
Aboveground total dry weight (**a–d**) and crop growth rate (**e–h**) in the early and late seasons of 2013 and 2014. Error bars represent ± 1 s.e. (n = 4, standard error of four replications).

Yield difference between ICM and FP was due to the difference in aboveground total dry weight (TDW) rather than in harvest index (HI) (Tables 4 and 5). The TDW of ICM at maturity was 13.9–38.9% higher than that of FP (Table 5). From tillering to flowering, the late-season rice exhibited larger difference between ICM and FP in TDW than the early-season rice (Fig. 2a–d). Across the entire growing season, ICM had consistently higher crop growth rate (CGR) than FP in three out of the four field experiments (Fig. 2e–h). Yield advantage of the late season over the early season was due to both TDW and HI in 2013, but was due to TDW alone in 2014 (Tables 4 and 5). Overall, the early-season rice in 2013 had the lowest grain filling percentage and HI among the four field experiments, which was due to high temperature stress during flowering (daily maximum temperature of 36.2–37.0 °C on June 17–19).

### Physiological characteristics

Maximum leaf area index (LAI) and leaf area duration (LAD) of ICM were significantly higher than those of FP (Table 4). The ICM treatment had higher LAI than FP throughout the growing season except for the vegetative stage in the early-season rice in 2014 (Supplementary Fig. 1). Higher LAI was responsible higher CGR and TDW in ICM compared with FP. The ICM treatment also had higher stems per m^2^ than FP throughout the growing season except for the vegetative stage in the early-season rice in 2014 (Supplementary Fig. 2). The maximum stems per m^2^ of ICM were higher than that of FP (Table 4). Higher panicles per m^2^ of ICM was attributed to higher stem number, which was due to higher plant density with narrower hill spacing and more seedlings per hill compared with FP.

The RUE of ICM was 13.6–35.0% higher than that of FP (Table 5). The differences between ICM and FP in light interception percentage and intercepted radiation were relatively small compared with the difference in TDW between the two treatments. Higher RUE was observed in the early-season rice than in the late-season rice. There was no consistent difference in RUE between 2013 and 2014 (Table 5).

### Nitrogen uptake and use efficiency

The ICM treatment has higher N uptake at maturity but lower N harvest index (NHI), N use efficiency for grain production, and partial factor productivity of applied fertilizer N than FP (Table 6). Agronomic N use efficiency was higher in ICM than in FP in the late-season rice, but no difference in the early-season rice. There was no consistent difference between ICM and FP in N recovery efficiency and physiological N use efficiency (Table 6), because no significant differences between ICM and FP were found in RE in the early season of 2014, and in PE in the late season of 2013. Overall, ICM tended to reduce NUE due to higher rate of N fertilizer application compared with FP. The early-season rice demonstrated higher N recovery efficiency than the late-season rice, but the reverse was true for NHI, N use efficiency for grain production, and physiological N use efficiency although the differences were relatively small in 2014 compared with in 2013 (Table 6). There was no consistent difference between the two seasons in other NUE-related traits.

## Discussion

On average, ICM produced the grain yield of 9.67 t ha^−1^ compared to 8.52 t ha^−1^ from FP, resulting in a 13.5% increase in grain yield over FP. Significant increase in grain yield by ICM over FP was also reported in single-season rice in China^10^,^11^,^21^,^22^,^23^, in double-season rice in China^12^, and in double-season rice in India^14^ and Bangladesh^9^. The higher grain yield of ICM was attributed to the increase in sink size, which was caused by more panicles per unit area compared with FP. It is interesting to observe that yield enhancement through improved crop management is generally realized through the increase in panicle number, while yield increase by varietal improvement is generally resulted from the large panicle size.

Higher biomass production instead of HI was responsible for higher grain yield of ICM over FP. The ICM had higher CGR than FP throughout the entire growing season in three out of four field experiments, which was associated with higher LAI, LAD, and RUE of ICM. Similar results were reported by Xue *et al*. and Qin *et al*. who attributed the yield gain of ICM to increased LAI and high radiation interception and RUE^12^,^21^. Notably, the RUE of ICM reached 2.01–2.12 g MJ^−1^ in the early-season rice and 1.49–1.76 g MJ^−1^ in the late-season rice in our study. These RUE values were similar to the potential values determined under high-yielding environment in previous studies^19^,^24^,^25^.

Increased plant density with narrower hill spacing and more seedlings per hill in ICM contributed to higher stem number per unit area and higher CGR during the vegetative phase compared with FP. Higher stem number per unit area was the prerequisite for higher panicle number at maturity in ICM. Peltonen-Sainio stated that improved early season growth capacity supported good establishment for high interception of solar radiation, which, in turn, determines total plant biomass and grain yield^26^.

Improvement in nutrient management in ICM with increased rates of N, P, and K application, and with more times of N and K application supported higher CGR throughout the growing season and higher N uptake and RUE as well compared with FP. Improved nutrient management in ICM with delayed N application was responsible for slower leaf senescence during ripening phase, as evidenced by higher ratio of flag leaf SPAD reading at maturity to that at flowering in ICM than FP (Supplementary Fig. 3). Slower leaf senescence of ICM could ensure the maintenance of higher LAI, CGR, RUE, and N uptake after flowering. Sui *et al*. also reported that N application at later reproductive growth stages had a benefit for grain yield, which might prevent and slow down leaf senescence, resulting in high photosynthetic activity^22^. Although the increased rates of N, P, and K application ensured that nutrients did not limit crop growth and yield formation in ICM, decline in nutrient use efficiency in ICM compared with FP would increase the risk of nutrient losses and cause environmental concerns.

The late-season rice produced 2.18 t ha^−1^ higher grain yield than the early-season rice in N0. The seasonal yield difference was reduced to 1.33–1.62 t ha^−1^ in FP and ICM. Yield difference was more than 1 t ha^−1^ between the two seasons in several provinces in central China^15^. As reported by Qin *et al*.^12^, sink size due to the different panicle size was mainly responsible for the yield difference between the two seasons. The lower grain yield in the early-season rice compared to the late-season rice could be partially attributed to varietal difference and different climatic conditions between the two seasons^12^. There was no doubt that lower temperature of the early-season rice reduced CGR during the vegetative phase, while higher temperature shortened the ripening phase by 14–21 d compared with the late-season rice. One strategy to overcome the limitation of low temperature on early vegetative growth in the early-season rice is to increase the rate of basal N application. However, high rate of N application at the early growth stage when the plant’s N uptake ability is still low could maximize the risk of N losses and reduce NUE. Both limited biomass accumulation during vegetative stage and shortened grain filling duration during gain development stage were detrimental to yield formation of the early-season rice. In addition, extremely high temperature may occur during flowering period in the early-season rice, which could induce spikelet sterility and reduce grain filling percentage and HI, and consequently lead to lower grain yield. This had happened in the early-season rice in 2013, as evidenced by lower grain filling percentage and HI compared with the other three field experiments (Tables 3 and 4). It appeared that the reduction in grain filling percentage and HI due to high temperature stress in 2013 early-season rice was more severe in ICM than in FP andN0, suggesting that caution should be taken when high nutrient input is used in ICM to enhance rice yield potential in high temperature-prone season or area.

Double-season rice generally has lower grain yield than single-season rice although its annual grain yield (i.e. summation of grain yield during both early and late seasons) is higher than the single-season rice^12^,^15^,^17^. Wu *et al*. stated that the attainable yield under double rice-cropping system is characterized by relatively lower grain yield of 5.46 t ha^−1^ in the early-season crop and 7.69 t ha^−1^ in the late-season crop^15^. Using data from on-farm experiments conducted in China’s major rice-producing regions from 2000 to 2013, Xu *et al*. reported average grain yields of 6.5, 8.0, and 6.9 t ha^−1^ for the early-, middle-, and late-season rice, respectively^17^. Under the best crop management treatment, Qin *et al*. was able to achieve 8.3 and 9.5 t ha^−1^ grain yield in the early- and late-season rice, respectively^12^. Similarly, a grain yield of 9.5 t ha^−1^ was produced by the hybrid cultivar Liangyou-287 in the early-season rice^27^ and by T-you 207 in the late season rice^28^. In our study, ICM achieved a maximum grain yield of 9.40 t ha^−1^ with hybrid cultivar Liangyou 287 in the early-season rice in 2014 and 10.53 t ha^−1^ with hybrid cultivar Tianyouhuazhan in the late-season rice in 2013. More importantly, daily grain yield in the main field of ICM was more than 100 kg ha^−1^ d^−1^ for both the early- and late-season rice crops. One of the criteria for “super” rice varieties in China is to produce 100 kg ha^−1^ d^−1^ in the main field excluding the period in the seedbed^29^. This is a plausible criterion because it eliminates the approach of improving yield potential by increasing crop growth duration so that cropping intensity could be maintained in the cropping system^30^. Daily grain yield is also an important criterion for judging the productivity of double-season rice crop due to limitation in total growth duration under subtropical conditions.

To achieve 9.0–10.5 t ha^−1^ grain yield in double-season rice, the following traits and their corresponding values should be considered: >45,000 spikelets m^−2^, >80% grain filling, >50% in HI, >1,700 g TDW m^−2^, >18 g m^−2^ d^−1^ in seasonal mean CGR, >7 in maximum LAI, >500 m^−2^ in maximum stem number, >70% in seasonal mean LI, >1.5 g MJ^−1^ in RUE, >200 kg N ha^−1^ in total N uptake, and >100 in kg ha^−1^ d^−1^ in daily grain yield. As suggested by Sui *et al*., it is difficult to increase rice yield potential by improving a single trait of yield component^22^. For example, increase in grain yield not only needs to enlarge sink size by increasing the number of panicles but also requires adjustment of other yield formation processes. The ICM was very effective in breaking the negative relationship among the yield-related traits and achieving an overall improvement in grain yield^22^.

In general, implementation of ICM involves in increased inputs in labor and resources^9^. Labor-demanding practices are less attractive to farmers as wages and the opportunity cost of labor are increasing with the progress in economic development^31^. Rice farmers in China are reluctant to invest more resources in the rice production because of lower rice prices^32^. The future research on ICM should consider the inclusion of labor-saving technologies, efficient nutrient management, and simplified crop management practices.

## Conclusions

Yield improvement of double-season rice with ICM was achieved with the combined effects of increased plant density and optimized nutrient management. A maximum grain yield of 9.40 and 10.53 t ha^−1^ was achieved under ICM in the early- and late-season rice, respectively, indicating the potential to further increase the grain yield of double-season rice following a holistic and integrated agronomic approach^12^. Yield gain of ICM resulted from a combination of increases in sink size due to more panicle number per unit area and biomass production, further supported by the increased LAI, LAD, RUE, CGR, and total N uptake compared with FP. Further enhancement in the yield potential of double-season rice should focus on increasing CGR and biomass production through improved and integrated crop management practices. There was a tendency that nitrogen use efficiency declined under ICM due to higher rate of nitrogen fertilizer application compared with FP. Therefore, future study should consider more efficient nutrient management in ICM.

## Materials and Methods

### Experiment design and plant materials

Experiments were conducted in 2013 and 2014 in a farmer’s field at Zhougan Village (29°51′N, 115°33′E, 51 m altitude), Dajin Township, Wuxue County, Hubei Province, China. In each year, rice was grown in a double-season cropping system with an early-season rice from March to July and a late-season rice from June to October or November. Detailed dates of sowing, transplanting, and maturity were given in Supplementary Table 1. The soil has the following properties: pH 5.1, 29.7 g kg^−1^ organic matter, 2.7 g kg^−1^ total nitrogen (N), 38.3 mg kg^−1^ Olsen phosphorus (P), and 301.8 mg kg^−1^ exchangeable potassium (K). The soil test was based on samples taken from the upper 20 cm of the soil before the application of basal fertilizers in 2013.

In each experiment, crop management treatments were arranged in a complete randomized block design with four replicates. Crop management treatments included N0, FP, and ICM. The differences in crop management practices among the three treatments were summarized in Supplementary Table 2. For the ICM treatment, we increased (1) the rates of N, P, and K application, (2) the times of N and K application, and (3) plant density with narrower hill spacing and more seedlings per hill compared with FP. High nutrient input was used to ensure that the yield potential of double-season rice was not limited by nutrient supply.

For FP, N as ammonium bicarbonate was applied at basal while N as urea was applied at midtillering. For both N0 and FP, P as superphosphate was applied at basal while K as KCl was applied at midtillering. For ICM, basal N application was consist of 84.0 kg N ha^−1^ as compound fertilizer and 119.7 kg N ha^−1^ as ammonium bicarbonate in the early season, and 78.8 kg N ha^−1^ as compound fertilizer and 53.8 kg N ha^−1^ as ammonium bicarbonate in the late season. Urea was used for topdressing in ICM in both seasons. Phosphorus was applied only at basal for ICM with 36.7 kg P ha^−1^ as compound fertilizer and 36.7 kg P ha^−1^ as superphosphate in the early season, and 34.4 kg P ha^−1^ as compound fertilizer and 16.5 kg P ha^−1^ as superphosphate in the late season. Potassium for ICM was applied at basal, midtillering, and panicle initiation. At basal, 69.7 and 65.3 kg K ha^−1^ as compound fertilizer was applied in the early and late seasons, respectively. Potassium chloride was used for K topdressing with 37.4 kg K ha^−1^ at midtillering and 74.7 kg K ha^−1^ at panicle initiation in the early season, and 87.4 kg K ha^−1^ at midtillering and 52.3 kg K ha^−1^ at panicle initiation in the late season. Zinc as zinc sulfate heptahydrate was applied only at basal for all treatments in both seasons.

The N0 treatment was embedded in the FP treatment. Plot sizes were 30, 90, and 120 m^2^ for N0, FP, and ICM, respectively. Varieties were Liangyou 287 for the early-season rice and Tianyouhuazhan for the late-season rice. Both varieties are F1 hybrids and widely grown for double-season rice crop in central China. Pre-germinated seeds were sown in nursery bed to produce uniform seedlings. Forty-three- to 45-day-old seedlings were manually transplanted for the early season, while 36-day-old seedlings were manually transplanted for the late season. A water depth of 5 to 10 cm was maintained until 7 days before maturity when the fields were drained. Weeds, insects, and diseases were controlled as required to avoid yield loss.

### Measurements

#### Plant sampling

Sowing, transplanting, panicle initiation, flowering, and maturity dates were recorded for determining crop growth duration. Twelve hills were sampled from each plot with an interval of 7–15 days during the growing season to measure stem number, leaf area index (LAI), and aboveground total dry weight (TDW). Plants were separated into green leaves and stems. Green leaf area was measured with a leaf area meter (LI-3000, LI-COR Inc., Lincoln, NE, USA) and expressed as LAI. Leaf area duration (LAD) was calculated as the product of LAI and duration in days. The dry weight of leaves and stems were determined after oven-drying at 70 °C to constant weight. Crop growth rate was calculated as the ratio of increases in TDW to the duration of growing period. Flag leaf SPAD value (Chlorophyll meter SPAD-502, Minolta, Ramsey, NJ) was measured at flowering and maturity stages. The ratio of SPAD at maturity to SPAD at flowering was used to quantify the rate of flag leaf senescence.

#### Yield and yield components

At maturity, 12 hills were taken diagonally from a 5 m^2^ area in each plot where grain yield was determined to measure LAI, TDW, harvest index (HI), and yield components. Panicles of each hill were counted to determine the panicle number per m^2^. Plants were separated into green leaves, stems, and panicles. Panicles of all 12 hills were hand threshed and filled spikelets were separated from unfilled spikelets by submerging them in tap water. Three subsamples with each of 30 g filled spikelets and 10 g unfilled spikelets were taken to determine the number of spikelets. Dry weights of green leaves, stems, rachis, and filled and unfilled spikelets were measured after oven drying at 70 °C to constant weight. Spikelets per panicle, grain filling percentage (100 × filled spikelets/total spikelets), and HI (100 × filled spikelet weight/TDW) were calculated. Grain yield was determined from a 5 m^2^ area in each plot and adjusted to the standard moisture content of 0.14 g H_2_O g^−1^ fresh weight. Grain moisture content was measured with a digital moisture tester (DMC-700, Seedburo, Chicago, IL, USA). Daily grain yield was calculated as the ratio of grain yield to growth duration from transplanting to maturity.

#### Nitrogen uptake and use efficiency

Tissue N concentration was measured at maturity. After dry weight measurement, different organs were ground using a mixer mill homogenizer (MM400, Retsch, Germany). Approximately 5.0 mg was used to measure N concentration using an NC analyzer (IsoPrime100 IRMS, Isoprime Ltd, UK). Nitrogen uptake at maturity, N harvest index (NHI), N use efficiency for grain production, agronomic N use efficiency, N recovery efficiency, physiological N use efficiency, partial factor productivity of applied fertilizer N were calculated in both seasons according to Peng *et al*.^33^.

#### Radiation interception and use efficiency

In both seasons, climate data (daily solar radiation, minimum temperature, and maximum temperature) were collected from the weather station located within 2 km from the experimental site. A data logger (CR800, Campbell Scientific Inc., Logan, Utah, USA) was used as the measurement and control module. A silicon pyranometer (LI-200, LI-COR Inc., Lincoln, NE, USA) and temperature/RH probe (HMP45C, Vaisala Inc., Helsinki, Finland) were used to measure total solar radiation and temperature, respectively. Daily solar radiation from transplanting to maturity was used to determine seasonal accumulated radiation. Canopy light interception was measured between 1100 and 1300 h with an interval of 7–15 days during the growing season with a line ceptometer (AccuPAR LP-80, Decagon Devices Inc., Pullman, WA, USA). In each plot, light intensity inside the canopy was measured by placing the light bar in the middle of two rows and at approximately 5 cm above the water surface. Light intensity above the canopy was recorded immediately after the light measurement inside the canopy. Light interception was calculated as the percentage of light intercepted by the canopy [100 × (light intensity above canopy**-**light intensity below canopy)/light intensity above canopy]. Estimates of seasonal LI were made by linear interpolation of instantaneous measurements of LI with respect to days after transplanting according to Muurinen and Peltonen-Sainio^34^. Seasonal intercepted radiation during the entire growing season from transplanting to maturity was the product of seasonal LI and accumulated solar radiation during this growing period. Radiation use efficiency was calculated as the ratio of biomass production to seasonal intercepted radiation from transplanting to maturity.

### Data analysis

Data were analyzed following analysis of variance^35^ and means of crop management treatments were compared based on the least significant difference test (LSD) at the 0.05 probability.

## Additional Information

**How to cite this article**: Wang, D. *et al*. Integrated crop management practices for maximizing grain yield of double-season rice crop. *Sci. Rep.* **7**, 38982; doi: 10.1038/srep38982 (2017).

**Publisher's note:** Springer Nature remains neutral with regard to jurisdictional claims in published maps and institutional affiliations.

## Supplementary Material

Supplementary Information

## Figures and Tables

**Table 1:** Climate conditions by crop growth stages for the early- and late-season rice in 2013 and 2014.

Year	Season	Min T^a^	Max T	RAD
		Transplanting to Panicle initiation		
2013	Early	17.8	26.2	13.9
	Late	26.8	35.4	20.6
2014	Early	16.3	25.8	14.7
	Late	24.1	31.3	13.7
		Panicle initiation to Flowering		
2013	Early	22.0	29.4	15.8
	Late	22.4	30.4	15.1
2014	Early	22.1	29.8	14.9
	Late	22.4	30.8	13.2
		Flowering to Maturity		
2013	Early	25.3	32.0	15.0
	Late	13.9	25.4	11.4
2014	Early	23.7	29.9	12.4
	Late	16.9	26.8	12.2
		Transplanting to Maturity		
2013	Early	21.9	29.3	15.0
	Late	19.7	29.5	14.9
2014	Early	20.9	28.5	13.7
	Late	20.1	29.0	12.9

^a^Average daily minimum temperature (Min T, °C), average daily maximum temperature (Max T, °C), and average daily solar radiation (RAD, MJ m^−2^ day^−1^) for each growing period.

**Table 2:** Growth duration (days) for the early- and late-season rice in 2013 and 2014.

Year	Season	SW to TR^a^	TR to PI	PI to FL	FL to MA	SW to MA	TR to MA
2013	Early	45	24	31	27	127	82
	Late	36	28	29	48	141	105
2014	Early	43	25	28	32	128	85
	Late	36	23	26	46	131	95

^a^SW, TR, PI, FL, and MA are sowing, transplanting, panicle initiation, flowering, and maturity, respectively.

**Table 3:** Yield components for the early- and late-season rice in 2013 and 2014.

Year	Season	Treat.	Panicles m^−2^	Spikelets panicle^−1^	Spikelets m^−2^ (×10^3^)	Grain filling (%)	1000-grain weight (g)
2013	Early	N0^a^	255.3^c^	108.6^b^	27.7^c^	81.2^a^	23.3^a^
		FP	308.5^b^	138.7^a^	42.8^b^	70.0^b^	22.2^c^
		ICM	498.8^a^	102.0^b^	50.8^a^	65.1^c^	22.7^b^
		Mean	354.2	116.4	40.4	72.1	22.7
	Late	N0	241.2^c^	166.8^a^	40.2^c^	82.3^a^	22.2^a^
		FP	289.5^b^	164.0^a^	47.5^b^	76.7^b^	22.4^a^
		ICM	395.7^a^	142.4^b^	56.3^a^	78.3^b^	22.4^a^
		Mean	308.8	157.8	48.0	79.1	22.3
2014	Early	N0	229.8^c^	88.6^c^	20.3^c^	92.2^a^	25.2^a^
		FP	341.6^b^	120.3^a^	41.1^b^	81.9^b^	24.1^b^
		ICM	397.2^a^	114.3^b^	45.4^a^	82.2^b^	24.1^b^
		Mean	322.8	107.7	35.6	85.4	24.5
	Late	N0	205.5^c^	166.5^a^	34.2^c^	84.2^a^	22.8^b^
		FP	244.8^b^	173.1^a^	42.3^b^	84.0^a^	22.9^b^
		ICM	377.4^a^	134.3^b^	50.7^a^	80.7^b^	23.2^a^
		Mean	275.9	158.0	42.4	83.0	22.9

Within a column for each season and year, means followed by the same letters are not significantly different according to LSD (0.05).

^a^N0, FP, and ICM are zero-N, farmers’ practice, and integrated crop management, respectively.

**Table 4:** Maximum leaf area index, leaf area duration, maximum stem number, harvest index, and daily grain yield for the early- and late-season rice in 2013 and 2014.

Year	Season	Treat.	Maximum leaf area index	Leaf area duration^a^ (m^2^ d m^−2^)	Maximum stem number m^−2^	Harvest index (%)	Daily grain yield (kg ha^−1^ d^−1^)
2013	Early	N0^b^	2.71^c^	134.3^c^	303.9^c^	46.0^b^	69.0^c^
		FP	4.56^b^	210.9^b^	364.7^b^	48.2^a^	89.9^b^
		ICM	7.91^a^	378.8^a^	544.8^a^	39.2^c^	101.5^a^
		Mean	5.06	241.4	404.4	44.5	86.8
	Late	N0	3.91^c^	198.4^c^	386.4^b^	54.1^a^	73.2^c^
		FP	5.00^b^	293.3^b^	413.4^b^	53.1^a,b^	84.6^b^
		ICM	8.00^a^	468.2^a^	691.2^a^	50.1^b^	100.3^a^
		Mean	5.64	320.0	497.0	52.4	86.0
2014	Early	N0	2.32^c^	117.6^c^	299.4^b^	55.4^a^	60.7^c^
		FP	5.52^b^	291.3^b^	466.3^a^	55.3^a^	98.1^b^
		ICM	6.82^a^	362.5^a^	517.2^a^	53.5^b^	110.6^a^
		Mean	4.89	257.1	427.6	54.8	89.8
	Late	N0	3.57^c^	199.9^c^	279.8^c^	55.8^a^	78.8^c^
		FP	5.46^b^	329.9^b^	364.2^b^	55.0^a^	99.9^b^
		ICM	8.11^a^	556.9^a^	530.5^a^	51.4^b^	109.8^a^
		Mean	5.71	362.2	391.5	54.0	96.2

Within a column for each season and year, means followed by the same letters are not significantly different according to LSD (0.05).

^a^Leaf area duration was calculated from transplanting to maturity.

^b^N0, FP, and ICM are zero-N, farmers’ practice, and integrated crop management, respectively.

**Table 5:** Seasonal solar radiation utilization for the early- and late-season rice in 2013 and 2014.

Year	Season	Treat.	Incident radiation^a^ (MJ m^−2^)	Intercepted percent (%)	Intercepted radiation (MJ m^−2^)	Total dry weight (g m^−2^)	Radiation use efficiency (g MJ^−1^)
2013	Early	N0^b^	1230.2	57.8^c^	710.6^c^	1136.0^c^	1.60^b^
		FP	1230.2	71.3^b^	877.4^b^	1376.1^b^	1.57^b^
		ICM	1230.2	73.4^a^	902.6^a^	1911.5^a^	2.12^a^
		Mean	1230.2	67.5	830.2	1474.5	1.76
	Late	N0	1564.4	72.7^c^	1136.5^c^	1354.4^c^	1.19^b^
		FP	1564.4	80.3^b^	1255.3^b^	1537.0^b^	1.22^b^
		ICM	1564.4	84.7^a^	1325.8^a^	1976.3^a^	1.49^a^
		Mean	1564.4	79.2	1239.2	1622.6	1.30
2014	Early	N0	1206.2	49.1^b^	591.6^b^	865.4^c^	1.47^c^
		FP	1206.2	70.1^a^	845.2^a^	1494.8^b^	1.77^b^
		ICM	1206.2	70.1^a^	845.5^a^	1702.2^a^	2.01^a^
		Mean	1206.2	63.1	760.8	1354.1	1.75
	Late	N0	1221.3	75.2^c^	918.3^c^	1185.0^c^	1.29^c^
		FP	1221.3	82.1^b^	1003.1^b^	1488.8^b^	1.48^b^
		ICM	1221.3	86.4^a^	1055.1^a^	1860.7^a^	1.76^a^
		Mean	1221.3	81.2	992.2	1511.5	1.51

Within a column for each season and year, means followed by the same letters are not significantly different according to LSD (0.05).

^a^Incident radiation, percent of intercepted radiation, intercepted radiation, aboveground total dry weight, and radiation use efficiency were calculated from transplanting to maturity.

^b^N0, FP, and ICM are zero-N, farmers’ practice, and integrated crop management, respectively.

**Table 6:** Nitrogen uptake and utilization for the early- and late-season rice in 2013 and 2014.

Year	Season	Treat.	N uptake^a^ (kg ha^−1^)	NHI (%)	NUE_g_ (kg kg^−1^)	AE (kg kg^−1^)	RE (%)	PE (kg kg^−1^)	PFP (kg kg^−1^)
2013	Early	N0^b^	109.4^c^	57.0^a^	47.8^a^	—	—	—	—
		FP	172.2^b^	57.8^a^	38.5^b^	7.2^a^	32.2^b^	22.3^a^	34.0^a^
		ICM	237.9^a^	46.8^b^	31.5^c^	9.2^a^	52.5^a^	17.4^b^	30.6^b^
		Mean	173.2	53.8	39.3	8.2	42.4	19.9	32.3
	Late	N0	114.9^c^	61.2^a^	63.7^a^	—	—	—	—
		FP	145.6^b^	64.3^a^	56.1^b^	4.4^b^	15.7^b^	26.2^a^	41.9^a^
		ICM	212.2^a^	54.5^b^	46.6^c^	9.9^a^	37.4^a^	26.4^a^	38.1^b^
		Mean	157.6	60.0	55.5	7.1	26.6	26.3	40.0
2014	Early	N0	89.6^c^	62.1^b^	53.5^a^	—	—	—	—
		FP	176.4^b^	63.2^a^	46.9^b^	17.8^a^	44.5^a^	39.9^a^	42.4^a^
		ICM	205.7^a^	59.1^c^	44.2^c^	17.6^a^	47.4^a^	37.1^b^	37.2^b^
		Mean	157.3	61.5	48.2	17.7	45.9	38.5	39.8
	Late	N0	128.4^c^	67.1^a^	51.4^a^	—	—	—	—
		FP	162.8^b^	69.1^a^	50.4^a^	8.1^b^	17.7^b^	49.4^a^	42.0^a^
		ICM	208.3^a^	55.1^b^	45.9^b^	11.3^a^	30.7^a^	36.9^b^	36.8^b^
		Mean	166.5	63.8	49.2	9.7	24.2	43.1	39.4

Within a column for each season and year, means followed by the same letters are not significantly different according to LSD (0.05).

^a^Nitrogen uptake at maturity, nitrogen harvest index (NHI), nitrogen use efficiency for grain production (NUE_g_), agronomic nitrogen use efficiency (AE), nitrogen recovery efficiency (RE), physiological nitrogen use efficiency (PE), partial factor productivity of applied fertilizer nitrogen (PFP).

^b^N0, FP, and ICM are zero-N, farmers’ practice, and integrated crop management, respectively.
