# Supplementary material for: Integrated crop management practices for maximizing grain yield of double-season rice crop
Source: Sci Rep. 2017 Jan 12;7:38982. doi: 10.1038/srep38982 (PMC5227689; doi:10.1038/srep38982)
Supplement: Supplementary Information [file srep38982-s1.pdf]

Supplementary information

Integrated crop management practices for maximizing grain yield of double-season rice crop

Depeng Wang, Jianliang Huang, Lixiao Nie, Fei Wang, Xiaoxia Ling, Kehui Cui, Yong Li, Shaobing Peng[*]

National Key Laboratory of Crop Genetic Improvement, MOA Key Laboratory of Crop Ecophysiology and Farming System in the Middle Reaches of the Yangtze River, College of Plant Science and Technology, Huazhong Agricultural University, Wuhan, Hubei 430070, China

Supplementary Table 1. Dates of field operations and crop growth stages for the early- and late-season rice in 2013 and 2014.

Year	Season	Sowing	Transplanting	Flowering	Maturity
2013	Early	13 Mar.	27 Apr.	21 Jun.	18 Jul.
	Late	17 Jun.	23 Jul.	18 Sep.	05 Nov.
2014	Early	15 Mar.	27 Apr.	19 Jun.	21 Jul.
	Late	19 Jun.	25 Jul.	12 Sep.	28 Oct.

Supplementary Table 2. Experimental treatments imposed for the early- and late-season rice in 2013 and 2014.

Season	Treat.	Fertilizer (kg ha^{-1}) (N−P−K−Zn)	N splits (kg ha^{-1}) (B−MT−PI)[a]	Spacing (cm)	Hills m^{-2}	Seedlings hill^{-1}
Early	N0[b]	0−39.3−74.7−6	−	16.7 × 20.0	30	2
	FP	196.5−39.3−74.7−6	127.5−69−0	16.7 × 20.0	30	2
	ICM[c]	244−73.4−181.8−6	203.7−24.2−16.1	10.0 × 26.7	37.5	4
Late	N0	0−39.3−74.7−6	−	16.7 × 26.7	22.5	2
	FP	196.5−39.3−74.7−6	127.5−69−0	16.7 × 26.7	22.5	2
	ICM[c]	261.6−50.9−205−6	132.6−80.7−48.3	10.0 × 26.7	37.5	4

[a]B, MT, and PI are the timing of N application at basal, midtillering, and panicle initiation, respectively.
[b]N0, FP, and ICM are zero-N, farmers' practice, and integrated crop management, respectively.
[c]Compound fertilizer (N-P_2O_5-K_2O, 15%-15%-15%) was applied as basal at 559.5 kg ha^{-1} for the early season and 525.0 kg ha^{-1} for the late season.

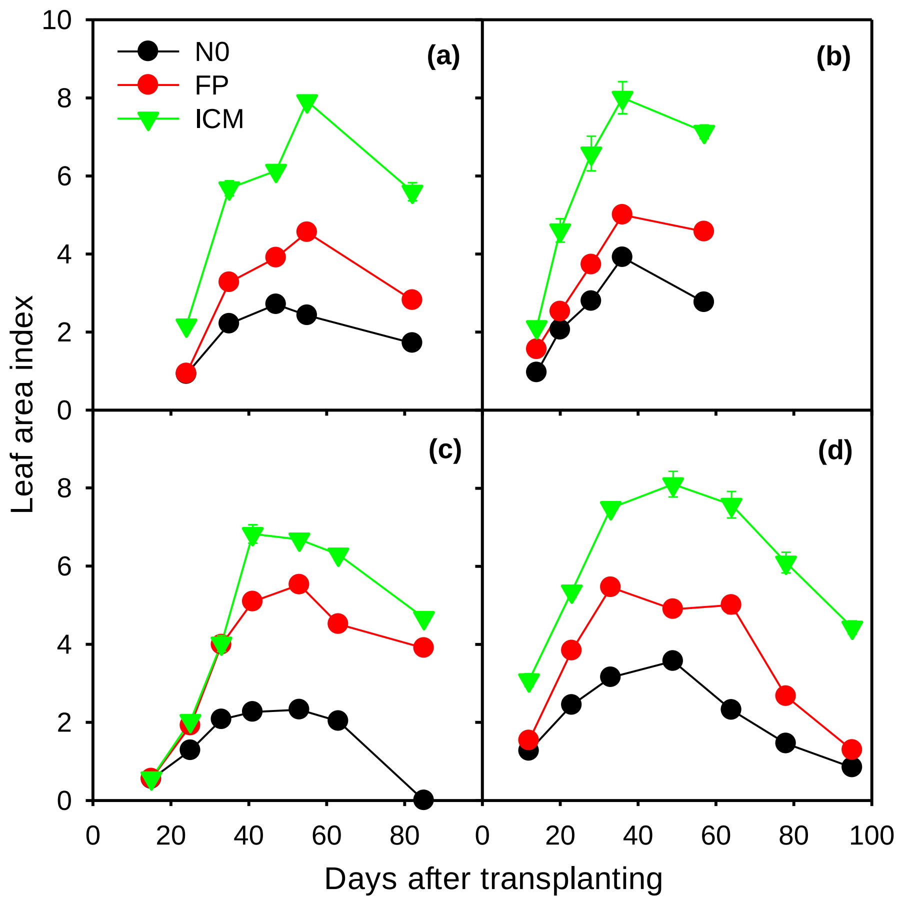

Supplementary Figure 1. Leaf area index in early (a) and late (b) seasons in 2013, and in early (c) and late (d) seasons in 2014. Error bars represent ±1 s.e. (n=4, standard error of four replications).

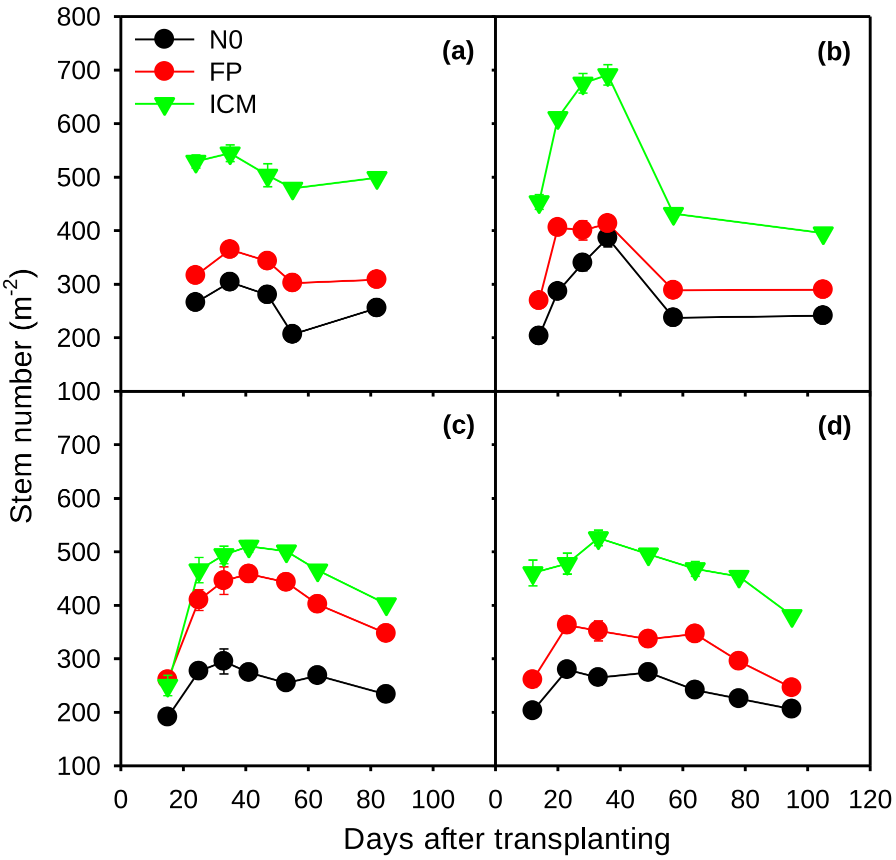

Supplementary Figure 2. Stem number per m^2 in early (a) and late (b) seasons in 2013, and in early (c) and late (d) seasons in 2014. Error bars represent ± 1 s.e. (n=4, standard error of four replications).

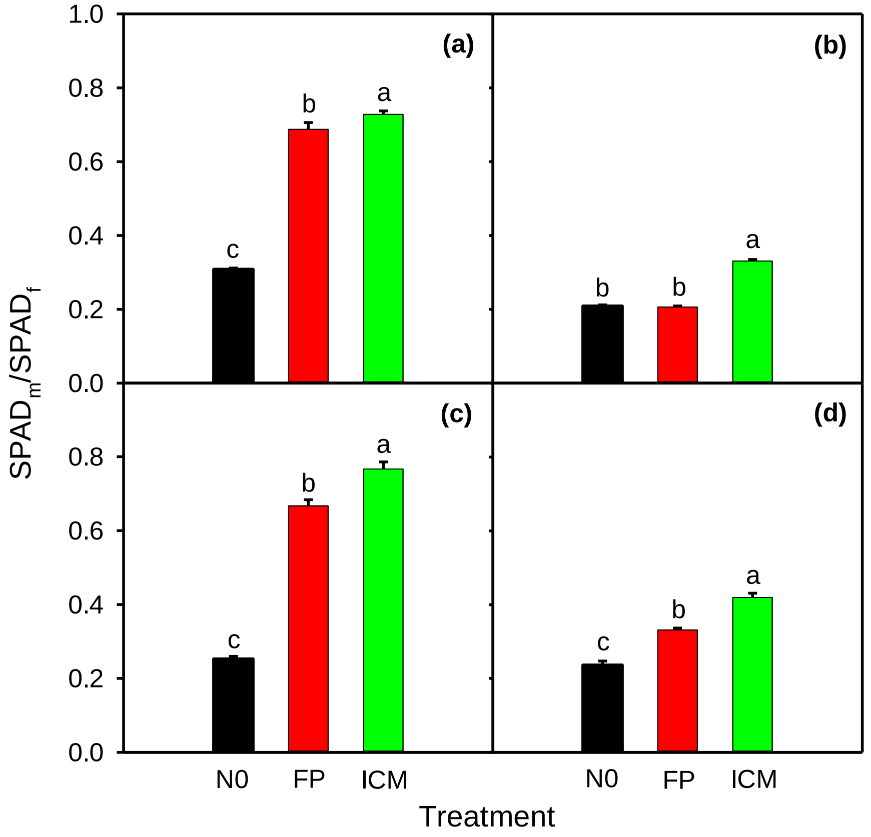

Supplementary Figure 3. The ratio of leaf SPAD value at maturity ($SPAD_m$) to leaf SPAD value at flowering ($SPAD_f$) in early (a) and late (b) seasons in 2013, and in early (c) and late (d) seasons in 2014. Different lowercase letters denote statistical differences between treatments of each season according to LSD test (0.05). Error bars represent ±1 s.e. (n=4, standard error of four replications).
